# Influence of Pre-Harvest Gibberellic Acid and Post-Harvest 1-methyl Cyclopropane Treatments on Phenolic Compounds, Vitamin C and Organic Acid Contents during the Shelf Life of Strawberry Fruits

**DOI:** 10.3390/plants10010121

**Published:** 2021-01-08

**Authors:** Akgül Taş, Selma Kuru Berk, Erdal Orman, Muttalip Gundogdu, Sezai Ercişli, Neva Karatas, Tunde Jurikova, Anna Adamkova, Sarka Nedomova, Jiri Mlcek

**Affiliations:** 1 Department of Plant and Animal Production, Seben İzzet Baysal Vocational School, Bolu Abant Izzet Baysal University, 14750 Seben Bolu, Turkey; akgultas1438@gmail.com; 2 Department of Plant and Animal Production, Mudurnu Sureyya Astarcı Vocational School, Bolu Abant Izzet Baysal University, 14800 Mudurnu Bolu, Turkey; selmakuru61@hotmail.com; 3 Atatürk Horticultural Central Research Institute, 77100 Yalova, Turkey; e.orman77@gmail.com; 4 Department of Horticulture, Faculty of Agriculture, Bolu Abant Izzet Baysal University, 14030 Bolu, Turkey; gundogdumuttalip@gmail.com; 5 Department of Horticulture, Faculty of Agriculture, Ataturk University, 25240 Erzurum, Turkey; sercisli@gmail.com; 6 Department of Nutrition and Dietetics, Faculty of Health Sciences, Ataturk University, 25240 Erzurum, Turkey; ngungor@atauni.edu.tr; 7 Institute for Teacher Training, Faculty of Central European Studies, Constantine the Philosopher University in Nitra, Dražovská 4, 949 74 Nitra, Slovakia; tjurikova@ukf.sk; 8 Department of Food Analysis and Chemistry, Faculty of Technology, Tomas Bata University in Zlín, 760 01 Zlín, Czech Republic; aadamkova@utb.cz; 9 Department of Food Technology, Faculty of AgriSciences, Mendel University in Brno, 613 00 Brno, Czech Republic; xnedomov@mendelu.cz

**Keywords:** strawberry, shelf-life, 1-MCP, GA_3_, biochemical compounds

## Abstract

In recent years, significant portions of the fresh fruits and vegetables produced worldwide have been decaying before reaching the consumer because of insufficient preservation after harvest. In this direction, we carried the study out to investigate the effect of gibberellic acid (GA_3_) and 1-methyl cyclopropane (1-MCP) applications on phenolic compounds and organic acid contents of the strawberry fruits (cv. Albion) during shelf-life. Gibberellic acid treatments, which prepared in two different concentrations (50 and 100 ppm), were performed by spraying the leaves before harvest. 1-methyl cyclopropane applied after harvest. The results of the study showed a greater decrease in organic acids (except oxalic and succinic acid) in Gibberellic acid-applied fruits during shelf-life. Citric acid was recorded as the most abundant organic acid in the control group. In phenolic compounds, gallic acid (15.22 mg 100 g^−1^) and ellagic acid (9.38 mg 100 g^−1^) were recorded as the highest phenolic compounds on the third day. 1-MCP and GA_3_ (50 ppm) + 1-MCP treatment reduced the breakdown of vitamin C during the shelf-life of strawberry fruits compared to the control group. As a result, phenolic compounds, vitamin C, and organic acids decreased during the shelf-life, and 1-MCP applications slowed down the breakdown of these compounds.

## 1. Introduction

Strawberry (*Fragaria x ananassa* Duch.) is a fruit, in the berry fruits, widely used in the food and cosmetics industry and for fresh consumption. Turkey ranks first in Europe with a production of 486,705 tonnes [1]. It ranks fifth in the world after China, the USA, Mexico, and Egypt [2]. Strawberry, which is important for human health, is a fruit rich in ascorbic acid, phenolic compounds, folates, sugars, and acids. Strawberry fruits are appetizing for people in terms of appearance and contain antioxidant compounds such as phenolic compounds and ascorbic acid, preventing free radicals from harming human health [3]. Additionally, this fruit with good taste and aroma has low calorie and high fiber content in terms of diet [3]. These compounds provide a synergistic and cumulative effect on protecting human health and disease (heart attack, anticarcinogen, etc.) prevention [4]. Phenolic compounds in vegetables, fruits, herbs, and beverages are a crucial constituent of the human diet [4]. Natural antioxidants are being preferred over synthetic antioxidants in recent years. With increasing demand in them with the preference of natural antioxidants, especially plant sourced, the importance of levels of these compounds in fruits became much more important [5,6]. These compounds have beneficial health effects such as hepatoprotection, anti-inflammation, and particularly antioxidation [7]. Besides being crucial antioxidants, they approve catechins as a positive regulation agent in epilepsy and epileptic disorder treatments [8]. Ellagic acid plays a crucial role in anti-inflammatory, anti-atherogenic, and neuroprotective effects [9].

Strawberry is sensitive to mechanical injuries and spoilage because it is soft textured. This situation shortens the shelf-life of the fruit [3,10]. Some fresh fruits and vegetables continue to ripen after harvest, and metabolic events such as ethylene production and respiration occur during this process. This process not only speeds up the maturation of the products but also affects its aroma, texture, and appearance, causing products to deteriorate and thus shorten the shelf-life [11,12]. Treatments to protect fruit quality criteria reduce moisture loss, color change, breathing, oxidation, and prolong shelf-life [13,14]. Fruits complete their physical development with the harvest. However, physiological events such as ethylene release and respiration continue after harvest. Thus, ripening in fruits speeds up, shelf-life shortened, and they affect the biochemical level of the fruit [11,15]. Strawberries known with rapid respiration characteristic after harvest and this harms shelf-life and fruit quality [16]. El-Kereamy et al. [17] reported that treatments with exogenous ethylene can accelerate the ripening of non-climacteric fruits. However, a clear relationship has not yet been established between ethylene and strawberry ripening. Indeed, in the study conducted by Manning [18], it was reported that ethylene inhibitors were not effective on the ripening of fruits. On the other hand, in the study conducted by El-Kazzaz et al. [19], it was noted that “G-3” and “G-4” strawberry cultivars exposed to ethylene formed a more intense red color and softened more quickly.

The composition of fruits and vegetables continues to change with the harvest. If there is no application for protection, this change shortens the shelf-life. In recent years, the use of 1-methyl cyclopropane (1-MCP) has become quite common to prolong the shelf-life of fruits and to minimize the change in the compositions. 1-MCP affects the functioning mechanism of ethylene and provides a delay in fruit ripening [20]. Additionally, it decreases the respiratory rate and prolongs the shelf-life of the fruit after harvest [21]. There are also studies that 1-MCP reduces the softening, browning, and rotting of ripe fruits [22]. Although the color of the fruit preserved with the 1-MCP application, the amount of pectin and total anthocyanin decrease with the softening of the flesh [23,24]. In studies conducted, pre-harvest applications also affected the preservation period of fruits. GA_3_ is among the plant growth regulators commonly used for this purpose. Plant growth regulators may stimulate or prevent, depending on the growth period of the plant and the genotype of the plant [25]. Gibberellins play an active role in cell division and elongation. Besides, plant growth regulators effective in root elongation, break apical rest, promote germination and seedless fruit formation when applied before flowering, and increase fruit weight when applied after flowering [26].

Plants produce more secondary metabolites under stress conditions. Phenolic compounds are one of the secondary metabolite groups that produced during their adaptation to abiotic conditions such as defense, protection, and maintaining their generations [27,28]. Recently, herbal polyphenols, which formed by the combination of flavonoids and non-flavonoid compounds, found as powerful antioxidants, neutralize the free radicals formed in the body, and prevent many diseases [29,30]. It also delays decay [31]. Therefore, it is important to protect phytochemicals such as phenolic compounds and organic acids in strawberry fruit after harvest. It is important to keep these secondary metabolites and nutritional values in healthy conditions during their shelf-life during the post-harvest period. In literature researches, research on the change in phenolic compounds and organic acid contents during the shelf-life of strawberry fruits observed limited. In this study, the effects of GA_3_ and 1-MCP treatments on the phenolic compound and organic acid contents of strawberry fruits were investigated during shelf-life.

## 2. Results and Discussion

### 2.1. Organic Acids and Vitamin C

In this study, the effects of GA_3_ and 1-MCP treatments on the organic acid composition of strawberry fruits during shelf-life examined. Results showed the treatments greatly affected the fruit’s organic acid properties. Similarly, increasing shelf-life had a significant effect on organic acid contents (*p* ≤ 0.05). Organic acid values decreased dramatically with the increase in shelf-life.

Citric acid was the pre-dominant organic acid in fruits and followed by malic, succinic, oxalic, and fumaric acid, respectively. The highest amount of oxalic acid in the shelf-life periods of the third (598.92 mg 100 g^−1^) and the fifth days (571.99 mg 100 g^−1^) observed in the 1-MCP application. The lowest amount of oxalic acid determined on the 5th day of the 100 ppm GA_3_ treatment (470.35 mg 100 g^−1^). In the study, citric acid content was determined as 14.95 mg 100 g^−1^ on the third day and 1425 mg 100 g^−1^ on the fifth day in the application of 1-MCP. In GA3 (50 ppm) treatment, it was recorded as 1346.96 mg 100 g^−1^ on the third day and 872.78 mg 100 g^−1^ on the fifth day. The minimum citric acid loss during the shelf-life was in the 1-MCP and the most occurred in the 100 ppm GA_3_ application. GA_3_ applications negatively affected malic acid content. The changes in tartaric acid content were like malic acid (Table 1). In comparison with the control group, 1-MCP mostly prevented the breakdown of organic acids except for fumaric and succinic acid. However, GA_3_ + 1-MCP prevented fumaric and succinic acid contents from decreasing more than the control group. In comparison with the control group, 1-MCP mostly prevented the breakdown of organic acids except for fumaric and succinic acid. However, GA_3_ + 1-MCP prevented fumaric and succinic acid contents from decreasing more than the control group. In the measurements made during the shelf-life of strawberry fruits, the lowest vitamin C content measured in 100 ppm GA_3_ treatment on the fifth day. When shelf-life periods compared, the decrease rate in the vitamin C content of the 5th-day fruits measured by approximately 50% (Table 2). In the study, 1-MCP application preserved the vitamin C content and prevents breakdown according to the control group. Compared with the control group, fruits treated with 1-MCP were found to reduce respiratory rate [32], reduce biochemical changes, and preserve organic acid and vitamin C content more. The positive effect of 100 ppm applications on organic acid change of fruits in GA_3_ applications has not been determined. When the fruits finish growing, ripening begins. It is a complex process in which maturation, softening and taste change and various metabolic activities take place [33]. The metabolic stages that take place in the maturation process require high energy, and this energy sourced from carbon compounds (sugars, amino acids, and organic acids) [34]. These metabolic changes a stage of accelerated ripening normally associated with increasing respiration [34]. The carbon source needed for the formation of soluble sugars in fruits; provided from starch, organic acids, and the cell wall. Since the amount of starch in strawberry fruit is low, organic acids and cell walls mostly used as a source and softening occurs in fruit flesh [35]. In this study, a significant decrease observed in organic acid levels in all treatments, supporting physiological notes about ripening. On the other hand, differences in reduction levels between treatments were not ruled out. Since strawberries do not continue to ripen after harvesting due to non-climacteric fruit formation, the fruits are harvested when they have a variety-specific size and color. Delay in harvest is understood by the softening of the fruit, losing its normal color and turning it darker. For distant markets, the harvest is done when three quarters of the fruit is browned. Well colored and shiny fruits are generally preferred in the market [36]. Exogenous application of plant growth regulators can promote fruit development [37]. Therefore, GA_3_ application, as a plant growth promoter, accelerates fruit development, and ripening. In the light of this information from the literature, the lowest organic acid in all applications of GA_3_ levels expected to detect, and it is believed that GA_3_ can contribute to the climacteric properties of the fruits as well as accelerating the harvest time. Unlike GA_3_, the 1-MCP application provided more stability in organic acid composition, supporting previous reports on the effects of 1-MCP [35,38]. This reveals that 1-MCP reduces breathing rate and ethylene synthesis, prevents fruit spoilage, and reduces the breakdown of organic acids [32]. In this study, it was reported that malic and citric acid contents of the “Dorit” and “Selva” strawberry cultivars decreased continuously during the storage similar to ours. Rahman et al. [3] reported that the highest titratable acidity is observed in 1/3 ripe fruits and decreases gradually as the maturity stage and storage period increase in all genotypes. In addition, the same researchers noted that the ascorbic acid content of strawberries gradually decreased during the storage period. It was determined that citric and malic acid levels was as 7.4 and 1.9 g L^−1^ in Benihoppe cv., respectively [39]. The researchers’ levels slightly higher than ours (14.95 g L^−1^ citric, and 0.80 g L^−1^ malic at most). The difference might occur because of cultivar differential.

**Figure 1:**
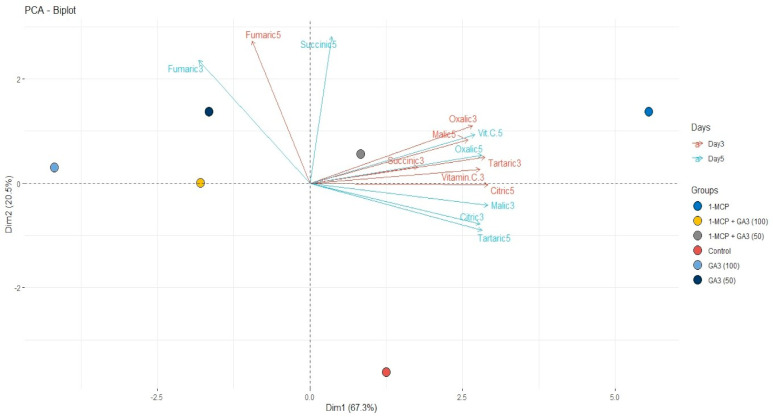
Distribution of organic acids, vitamin C, and treatments on the biplot.

The PCA analysis performed to determine relations between treatments, times, and organic acids. As a result, the first two components described almost 90% of the data (Figure 1). As a result of the PCA analysis, it was determined that the effective treatment was 1-MCP and it was located in the same PCA plane with the combination treatment with a 50 ppm dose of gibberellic acid. It was observed that the control group and GA_3_ treatments were in the same PCA plane and in the negative region compared to other treatments. Factor analysis also showed the first two components are enough for the explanation (*p* < 0.001). Citric, tartaric, malic, and succinic acid had almost the same amount of effect on PC1 (0.44 to 0.46) and described by them. PC2 described by fumaric acid (0.75) and vitamin C (0.42) contradicting PC1. The results also showed close relations between malic, tartaric, and oxalic acids. Furthermore, the 1-MCP and 1-MCP + GA_3_ (50) separated with only the amounts of these organic acids, although they distinguished with these from others. Another interesting detail PCA showed was between 100 ppm doses of GA_3_, which was similar to 1-MCP and 1-MCP + GA_3_ (50) but in the reverse direction. Overall, the control group clearly separated from all treatments (Figure 1).

### 2.2. Phenolic Compounds

In the study, the effects of treatments on changes in the specific phenolic compounds of fruits examined during shelf-life. The treatments caused statistically significant differences in the number of phenolic compounds (*p* ≤ 0.05). The decrease in phenolic compounds on the fifth day found to be approximately 50% in comparison with the third day. However, the decrease in ferulic acid ratio recorded less. In this study, the breakdown of phenolic compounds in fruits treated with 1-MCP found less than in other applications. Considering the changes of phenolic compounds during their shelf-life, on the third day, the highest gallic acid (11.43 mg 100^−1^), catechin (13.29 mg 100^−1^), and ellagic acid (9.38 mg 100^−1^) values were recorded in 1- MCP treatment. Protocatechuic acid found at the lowest rate compared to other phenolics (Table 3, Table 4 and Table 5). Among treatments, 1-MCP was more effective during the shelf-life (on the third day: 9.38 mg 100^−1^ and the fifth day: 7.80 mg 100^−1^) in preventing the degradation of ellagic acid, which has an anti-carcinogenic effect [39]. Although the content of ellagic acid varies from plant to plant, the species in the genus including strawberries known as the fruits with the highest ellagic acid content [40]. A report on ellagic acid content of raspberries showed ellagic levels vary between 270 mg 100 g^−1^ (wild raspberries) to 1900 mg 100 g^−1^(yellow raspberries) [41]. In our study, ellagic acid levels varied between 5.14 (GA_3_ (100)) and 9.38 mg 100 g^−1^ (1-MCP) that was significantly lower than in raspberries. It was noted that 1-MCP treatment during the shelf life prevented phenolic compounds loss of strawberry fruits more than the control group. In the study, the same changes recorded in other phenolic compounds. Pre-harvest gibberellic acid applications generally caused a decrease in gallic acid, protocatechuic acid, chlorogenic acid, caffeic acid, vanillic acid, ellagic acid, p-coumaric acid, and phloridzin contents during shelf-life. However, according to the control group, the disintegration of catechin and routine contents was found less during shelf-life. The application of 50 ppm GA_3_ had a positive effect on protecting ferulic acid and quercetin content, but the treatment of 100 ppm GA_3_ decreased the same compounds. The anthocyanin and phenolic compounds in berry fruits differ mostly depending on the species, variety, ripening stage, region, cultivation, and ecological conditions. Among these compounds, quercetin is the most common in flavonoids [42]. Caffeic and ferulic acids are the most common ones among many phenolic acids [43]. Both quercetin and ferulic acid exhibit crucial biological activities such as anti-inflammation and antioxidant, but one of the most crucial effects is the antioxidant capacity by scavenging reactive oxygen species (ROS) and enhancement of lipid oxidation related free radical [44].

**Figure 2:**
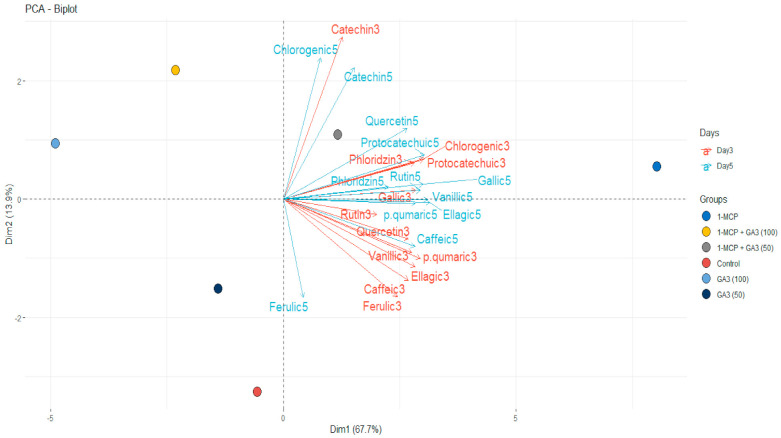
Distribution of phenolic compound contents of strawberry fruits during its shelf-life.

It was observed that there was a positive correlation between chlorogenic acid and catechin. The same correlation was found between quercetin, protocatechuic acid, chlorogenic, rutin, vanillic and gallic acid. When the distribution of phenolic compounds according to the Biplot graph examined, there was a negative relationship between catechin and ferulic acid. In this study, the first two principal components, which are the most important, explained 81.6% of the total variability for phenolic compounds of strawberry fruits during shelf-life (Figure 2). In the factor analysis, the first two components were sufficient to explain the variability (*p* < 0.001). Caffeic and vanillic acid had the highest effect on PC1 having almost the same amount of effect (0.43). PC2 mostly explained by chlorogenic acid (0.42) (Figure 2).

Allende et al. [45] noted that UV-C application caused a decrease in the amount of phenolic substance the increasing during storage time [45]. In some studies, the distribution and amount of some phenolic compounds in plants affected by factors such as maturity stage, cultivar, cultural applications, origin, growing season, storage conditions after harvest, processing, and plant growth regulators [46,47,48,49,50,51]. Besides, pre- and post-harvest applications of plant growth regulators affect fruit quality [52]. In another study, the effects of GA_3_ applications before harvest on the chemical and biochemical composition of “Obilnaja” Japanese plum fruits were limited [53].

## 3. Materials and Methods

### 3.1. Plant Material and Trial Pattern

**Figure 3:**
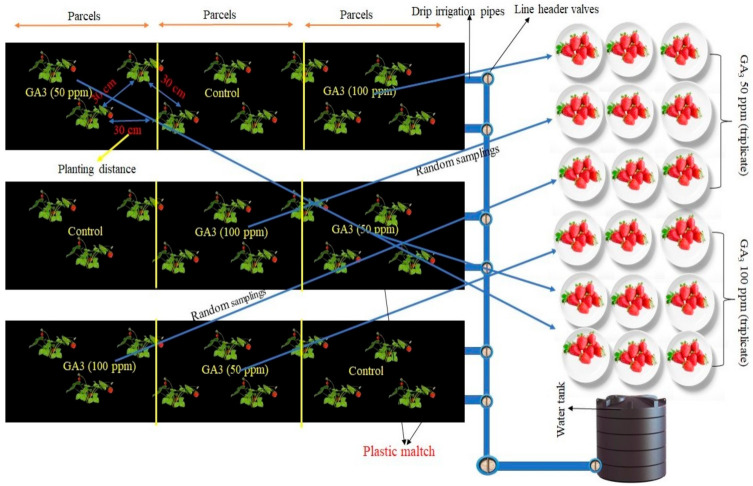
A summarized scheme of the field trial. The same procedure was implemented for the control group.

**Figure 4:**
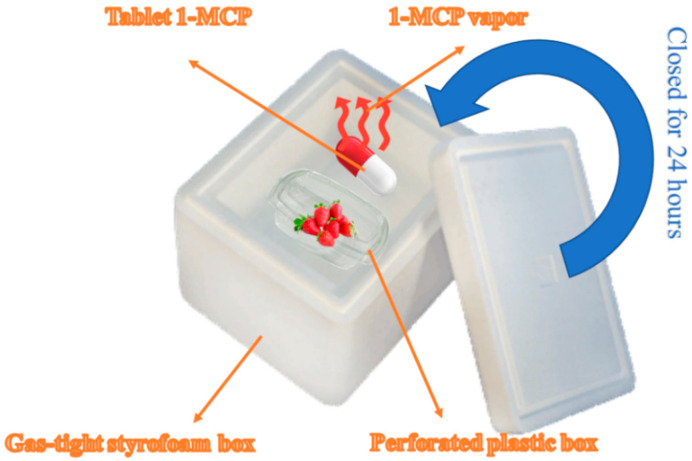
Preparation of 1-MCP and its application to fruits.

In this study, treatment parcels with frigo seedlings of Albion cv. established in the Bolu (Turkey) province. Seedlings planted in rows covered with black mulch and a drip irrigation system. Experiments conducted in randomized parcels design with 3 replications with 15 plants in each replication. Seedlings planted over seedling beds at 30 × 30 cm planting distance in a triangular sowing pattern. Seedling beds covered with black plastic mulching and irrigations performed through drip lines (Figure 3). Foliar GA_3_ spray treatments performed two times at 50 and 100 ppm doses. Kumar et al. [54] reported that 50, 100 and 150 ppm GA_3_ dozes were applied and the most effective results were obtained from 50 ppm treatment. Therefore, GA_3_ doses were used in this study by Kumar et al. [54]. The first application of GA_3_ was starting in bloom, and the second was 2 weeks after the first application. No application implemented in the control group. The fruit samples that came to harvest maturity collected by hand and carried to the laboratory in cloth bags. Smartfresh^®^ tablets used in the 1-MCP (0.625 ppb) application. The fruits brought to the laboratory placed in perforated plastic boxes. Then, these boxes placed in a gas-tight styrofoam box and 1-MCP applied at 20 °C for 24 h (Figure 4). 1-MCP application and the control groups’ fruits preserved on 22 °C for 3 and 5 days, and organic acids, vitamin C, and phenolic compounds contents of fruits measured on these days.

### 3.2. Determination of Phenolic Compounds

Phenolic compounds detected with a modified HPLC procedure suggested by Rodriguez-Delgado et al. [55]. Phenolic compound standards used in research: gallic and protocatechic standards were prepared at the levels of 20, 40, 60, 80, 100 and 120 ppm; catechin and ellagic standards were prepared at the levels of 500, 1000, 1500, 2000 and 2500 ppm; chlorogenic acid standard was prepared at the levels of 200, 400, 600, 800, 1000 and 1200 ppm; caffeic acid, quercetin and phloridzin standards were prepared at the levels of 100, 200, 300, 400, 500, and 600 ppm; vanillic acid, rutin, ferulic and p- qumarik standards were prepared at the levels of 50, 100, 150, 200, 250 and 300 ppm and formed curves. Fruit extracts mixed with distilled water in a ratio of 1:1. The mixture was centrifuged for 15 min at 15,000 rpm. Supernatants filtrated with coarse filter paper and twice with 0.45 µm membrane filter (Millipore Millex-HV Hydrophilic PVDF, Millipore, Taufkirchen, Germany), and injected into an HPLC (Agilent Technologies, Waldbronn, Germany). Chromatographic separation performed with a 250 × 4.6 mm, 4 μm ODS column (HiChrom, Bergenfield, NJ, USA). As mobile phase solvent A methanol: acetic acid: water (10:2:28) and Solvent B methanol: acetic acid: water (90:2:8) used. Spectral measurements made at 254 and 280 nm, and flow rate and injection volume adjusted to 1 mL min^−1^ and 20 µL, respectively.

### 3.3. Determination of Organic Acids

Organic acids identified by the technique reported by Bevilacqua and Califano [56]. Organic acid standards used in research; oxalic acid, tartaric acid, malic acid, citric acid and fumaric acid standards were prepared at the levels of 100, 200, 300, 400, 500, 600, 700 and 800 ppm and formed curves. Each sample (50 g) mixed with 80 mL of 0.009 N H_2_SO_4_ (Heidolph Silent Crusher M, Berlin, Germany), then homogenized for 1 h with a shaker (Heidolph Unimax 1010, Berlin, Germany). The mixture centrifuged for 15 min at 15,000 rpm, and supernatants filtrated twice with 0.45 µm membrane filter following filtration with coarse filter (Millipore Millex-HV Hydrophilic PVDF, Millipore, Burlington, MA, USA) and run through a SEP-PAK C18 cartridge. Organic acid readings performed with HPLC using Aminex column (HPX—87 H, 300 mm × 7.8 mm, Bio-Rad Laboratories, Richmond, CA, USA) at 214 and 280 nm wavelengths, on Agilent package program (Agilent, Santa Clara, CA, USA).

### 3.4. Determination of Vitamin C

Vitamin C content of fruits detected with a modified HPLC procedure suggested by Cemeroglu [57]. Fruit extracts (50 g) supplemented with 2.5% (w v^−1^) metaphosphoric acid (Sigma, M6285, 33.5%, Taufkirchen, Germany), then centrifuged at 6500 rpm for 10 min at 4 °C temperature. 0.5 mL of the mixture brought to a final volume of 10 mL with % 2.5 (w v^−1^) metaphosphoric acid. Supernatants filtered with a 0.45 μm PTFE syringe filter (Millex-HV Hydrophilic PVDF, Millipore, Taufkirchen, Germany). C18 column (Phenomenex Luna C18, 250 × 4.60 mm, 5 µL) was used for the identification of ascorbic acid at a temperature of 25 °C. Double-distilled water with 1 mL min^−1^ flow rate and pH of 2.2 (acidified with H_2_SO_4_) used as a mobile phase. Spectral measurements made at 254 nm wavelength using a DAD detector. Different standards of Vitamin C (Sigma A5960) (50, 100, 500, 1000, and 2000 ppm) used for quantification.

### 3.5. Statistical Analysis

The introductory statistics belonging to analysis and measurement results presented as average ± standard deviation. Experiments conducted in randomized parcels design with 3 replications with 15 plants in each replication. In this study, 3 different parallel analyzes were made except for replications. Analysis of variance was used to determine the differences between treatments’ averages in terms of phenolic compounds, organic acids, and vitamin C. The Duncan test was used as a multiple comparison test to express the differences between the averages. In the statistical evaluations, Windows SPSS 20 used, and the differences between the means evaluated by subjecting to ANOVA variance analysis and determined with Duncan multiple comparison tests (*p* < 0.05). The principal component analysis (PCA) was used to determine the correlation between treatments, traits, and shelf-life. Principal Component Analysis (PCA) biplot graph performed with The Comprehensive R Archive Network [58].

## 4. Conclusions

One of the goals of storage is to provide optimum quality criteria by minimizing changes in the color, aroma, physical, and biochemical structure of fruits and vegetables. In this study, the effect of GA_3_ and 1-MCP treatments on organic acid, vitamin C, and phenolic compound contents of Albion fruits found statistically significant. The effect of the treatments varied according to the organic acid and phenolic substances. Besides, the shelf-life was effective on organic acid, vitamin C, and phenolic compound amounts. In this study, it was determined that 1-MCP applications, which cause slowing down of physiological changes by decreasing the respiratory rate, prevent the degradation of organic acids and phenolic compounds in strawberry fruits. Generally, a decrease in the organic acid contents detected in fruits treated with gibberellic acid. The organic acid contents of fruits with 1-MCP applied determined to higher than the control group. Many researchers reported that 1-MCP slows down fruit ripening after harvest and therefore the conversion of organic acids into sugars. In the present study, a decrease in phenolic compounds determined as shelf-life increases. Additionally, 1-MCP treatment, which slows down the ripening of the fruit after harvest, prevented the breakdown and reduction of phenolic compounds more than other treatments. Studies on phytochemical changes that occur during the shelf-life of fruits limited. Therefore, this study thought to be an important guide and milestone for studies on strawberries shelf-life.

## Figures and Tables

**Table 1:** Effects of 1-MCP and GA_3_ on oxalic, citric, tartaric, and malic acid contents during shelf-life of strawberry fruits (mg 100 g^−^^1^).

	Treatments	Shelf-Life (Days)	
		3	5
Oxalic acid	Control	535.99 ± 5.00 c *	521.45 ± 0.50 b
	1-MCP	598.92 ± 1.00 a	571.99 ± 1.00 a
	GA_3_ (50)	543.83 ± 2.00 c	522.20 ± 1.50 b
	1-MCP + GA_3_ (50)	578.27 ± 0.50 b	520.61 ± 2.00 b
	GA_3_ (100)	510.66 ± 1.00 cd	470.35 ± 1.50 d
	1-MCP + GA_3_ (100)	526.42 ± 0.50 c	512.56 ± 1.00 c
Citric acid	Control	1470.39 ± 4.50 b	1085.42 ± 2.50 b
	1-MCP	1495.77 ± 0.50 a	1425.13 ± 0.50 a
	GA_3_ (50)	1346.96 ± 1.50 c	872.78 ± 1.00 c
	1-MCP + GA_3_ (50)	1390.47 ± 1.00 d	948.66 ± 1.00 d
	GA_3_ (100)	1204.05 ± 0.50 f	711.45 ± 1.00 f
	1-MCP + GA_3_ (100)	1291.45 ± 0.50 e	745.51 ± 1.00 e
Tartaric acid	Control	224.40 ± 3.00 ab	190.80 ± 0.50 b
	1-MCP	256.75 ± 1.50 a	220.50 ± 0.50 a
	GA_3_ (50)	216.54 ± 1.50 b	92.53 ± 0.40 e
	1-MCP + GA_3_ (50)	220.79 ± 1.50 b	148.02 ± 0.50 c
	GA_3_ (100)	207.88 ± 1.00 c	80.48 ± 0.50 f
	1-MCP + GA_3_ (100)	215.10 ± 0.50 bc	131.47 ± 0.50 d
Malic acid	Control	739.99 ± 5.00 b	323.90 ± 0.50 d
	1-MCP	803.77 ± 0.50 a	553.77 ± 3.00 a
	GA_3_ (50)	660.60 ± 0.50 e	284.15 ± 1.50 e
	1-MCP + GA_3_ (50)	692.55 ± 1.00 c	392.31 ± 1.00 c
	GA_3_ (100)	638.04 ± 0.50 f	254.10 ± 1.00 f
	1-MCP + GA_3_ (100)	673.45 ± 1.00 d	397.13 ± 1.50 b

*: Values are mean value ± standard deviation. *n* = 3. The difference among the means indicated with the same lower-case letter in columns was not significant (*p* ≤ 0.05).

**Table 2:** Effect of 1-MCP and GA_3_ treatments on the succinic acid, fumaric acid, and vitamin C contents during shelf-life of strawberry fruits (mg 100 g^−1^).

	Treatments	Shelf-Life (Days)	
		3	5
Succinic acid	Control	646.05 ± 5.00 d *	345.58 ± 1.00 e
	1-MCP	667.60 ± 0.50 c	612.03 ± 1.00 c
	GA_3_ (50)	672.62 ± 0.50 b	640.91 ± 0.50 a
	1-MCP + GA_3_ (50)	681.91 ± 1.00 a	620.04 ± 1.00 b
	GA_3_ (100)	572.21 ± 0.50 e	528.65 ± 0.50 d
	1-MCP + GA_3_ (100)	526.90 ± 1.00 f	473.82 ± 1.50 f
Fumaric acid	Control	2.59 ± 0.02 f	1.28 ± 0.14 f
	1-MCP	6.32 ± 0.30 e	4.63 ± 0.08 c
	GA_3_ (50)	9.76 ± 0.05 a	5.17 ± 0.07 a
	1-MCP + GA_3_ (50)	7.42 ± 0.03 d	3.60 ± 0.06 e
	GA_3_ (100)	9.48 ± 0.03 b	5.10 ± 0.02 b
	1-MCP + GA_3_ (100)	9.15 ± 0.07 c	4.58 ± 0.36 d
Vitamin C	Control	27.12 ± 0.50 c	16.99 ± 0.30 d
	1-MCP	33.79 ± 0.03 a	19.93 ± 0.06 a
	GA_3_ (50)	23.48 ± 0.40 e	16.61 ± 0.03 d
	1-MCP + GA_3_ (50)	30.47 ± 0.06 b	17.50 ± 0.27 b
	GA_3_ (100)	20.36 ± 0.10 f	16.57 ± 0.04 d
	1-MCP + GA_3_ (100)	26.88 ± 0.07 d	16.91 ± 0.08 c

*: Values are mean value ± standard deviation. *n* = 3 for organic acids and vitamin C. The difference among the means indicated with the same lowercase letter in columns was not significant (*p* ≤ 0.05).

**Table 3:** Effect of 1-MCP and GA_3_ treatments on the gallic, protocatechuic, catechin, and chlorogenic acid contents during shelf-life of strawberry fruits (mg 100 g^−1^).

	Treatments	Shelf-Life (Days)	
		3	5
Gallic Acid	Control	9.72 ± 0.04 c *	7.65 ± 0.15 b
	1-MCP	11.43 ± 0.05 a	10.05 ± 0.05 a
	GA_3_ (50)	9.53 ± 0.49 c	6.93 ± 0.49 c
	1-MCP + GA_3_ (50)	11.08 ± 0.05 b	7.50 ± 0.05 b
	GA_3_ (100)	8.96 ± 0.01 e	6.72 ± 0.05 d
	1-MCP + GA_3_ (100)	9.03 ± 0.00 d	7.45 ± 0.04 b
Protocatechuic Acid	Control	0.23 ± 0.02 c	0.17 ± 0.05 d
	1-MCP	0.44 ± 0.01 a	0.39 ± 0.06 a
	GA_3_ (50)	0.21 ± 0.00 d	0.13 ± 0.01 e
	1-MCP + GA_3_ (50)	0.35 ± 0.03 b	0.25 ± 0.025 b
	GA_3_ (100)	0.15 ± 0.04 e	0.10 ± 0.02 f
	1-MCP + GA_3_ (100)	0.24 ± 0.00 c	0.19 ± 0.00 c
Catechin	Control	7.52 ± 0.23 f	6.89 ± 0.23 e
	1-MCP	13.29 ± 0.11 a	11.72 ± 0.05 a
	GA_3_ (50)	9.01 ± 0.99 e	8.33 ± 5.00 d
	1-MCP + GA_3_ (50)	11.21 ± 0.05 d	9.39 ± 0.05 c
	GA_3_ (100)	11.62 ± 0.03 c	10.28 ± 0.05 b
	1-MCP + GA_3_ (100)	11.81 ± 0.44 b	9.27 ± 0.01 c
Chlorogenic Acid	Control	4.03 ± 0.48 c	2.65 ± 0.10 e
	1-MCP	4.89 ± 0.01 a	3.17 ± 0.04 c
	GA_3_ (50)	3.96 ± 1.00 d	3.01 ± 1.00 c
	1-MCP + GA_3_ (50)	4.35 ± 0.05 b	3.61 ± 0.05 a
	GA_3_ (100)	3.93 ± 0.03 d	2.91 ± 0.10 d
	1-MCP + GA_3_ (100)	4.02 ± 0.00 c	3.35 ± 0.03 b

*: Values are mean value ± standard deviation. *n* = 3 for the phenolic compounds. The difference among the means indicated with the same lowercase letter in columns was not significant (*p* ≤ 0.05).

**Table 4:** Effect of 1-MCP and GA_3_ treatments on the caffeic, vanillic, rutin, and ellagic acid contents during shelf-life of strawberry fruits (mg 100 g^−1^).

	Treatments	Shelf-Life (Days)	
		3	5
Caffeic acid	Control	1.37 ± 0.04 b *	0.919 ± 0.18 b
	1-MCP	1.55 ± 0.06 a	1.275 ± 0.02 a
	GA_3_ (50)	0.81 ± 0.19 d	0.56 ± 0.34 c
	1-MCP + GA_3_ (50)	0.95 ± 0.03 c	0.60 ± 0.03 c
	GA_3_ (100)	0.44 ± 0.03 f	0.41 ± 0.05 d
	1-MCP + GA_3_ (100)	0.72 ± 0.00 e	0.59 ± 0.03 c
Vanillic acid	Control	1.21 ± 0.10 b	0.86 ± 0.05 b
	1-MCP	1.50 ± 0.05 a	1.28 ± 0.05 a
	GA_3_ (50)	0.91 ± 0.05 c	0.70 ± 0.20 c
	1-MCP + GA_3_ (50)	0.94 ± 0.01 c	0.89 ± 0.01 b
	GA_3_ (100)	0.86 ± 0.02 c	0.64 ± 0.03 d
	1-MCP + GA_3_ (100)	0.88 ± 0.00 c	0.72 ± 0.05 c
Rutin	Control	1.51 ± 0.24 c	0.80 ± 0.01 c
	1-MCP	1.92 ± 0.04 a	1.29 ± 0.04 a
	GA_3_ (50)	1.80 ± 0.35 b	0.91 ± 0.19 b
	1-MCP + GA_3_ (50)	1.94 ± 0.01 a	0.84 ± 0.04 bc
	GA_3_ (100)	1.56 ± 0.03 c	0.74 ± 0.03 cd
	1-MCP + GA_3_ (100)	1.27 ± 0.00 d	0.80 ± 0.05 c
Ellagic acid	Control	8.21 ± 0.05 b	5.27 ± 0.18 bc
	1-MCP	9.38 ± 0.02 a	7.80 ± 0.13 a
	GA_3_ (50)	7.53 ± 2.49 c	5.33 ± 0.49 bc
	1-MCP + GA_3_ (50)	7.76 ± 0.02 c	5.77 ± 0.05 b
	GA_3_ (100)	5.14 ± 0.05 e	4.23 ± 0.02 cd
	1-MCP + GA_3_ (100)	6.58 ± 0.03 d	4.77 ± 0.00 c

*: Values are mean value ± standard deviation. *n* = 3 for the phenolic compounds. The difference among the means indicated with the same lowercase letter in columns was not significant (*p* ≤ 0.05).

**Table 5:** Effect of 1-MCP and GA_3_ treatments on the *p*-qumaric acid, ferulic, phloridzin, and quercetin acid contents during shelf-life of strawberry fruits (mg 100 g^−1^).

	Treatments	Shelf-Life (Days)	
		3	5
*p*-qumaric	Control	7.57 ± 0.31 b *	5.10± 0.04 c
	1-MCP	8.62 ± 0.03 a	6.28 ± 0.05 a
	GA_3_ (50)	6.51 ± 0.50 d	4.01 ± 1.00 d
	1-MCP + GA_3_ (50)	7.05 ± 0.00 c	5.84 ± 0.01 b
	GA_3_ (100)	5.75 ± 0.04 f	3.36 ± 0.03 e
	1-MCP + GA_3_ (100)	6.07 ± 0.00 e	4.30 ± 0.05 d
Ferulic acid	Control	1.43 ± 0.05 ab	1.37 ± 0.09 a
	1-MCP	1.58 ± 0.05 a	1.41 ± 0.03 a
	GA_3_ (50)	1.51 ± 0.05 a	1.53 ± 0.50 a
	1-MCP + GA_3_ (50)	1.36 ± 0.01 bc	1.35 ± 0.03 a
	GA_3_ (100)	1.17 ± 0.06 c	1.46 ± 0.10 a
	1-MCP + GA_3_ (100)	1.20 ± 0.00 c	0.95 ± 0.04 b
Phloridzin	Control	0.66 ± 0.44 bc	0.54 ± 0.04 c
	1-MCP	0.97 ± 0.04 a	0.67 ± 0.03 a
	GA_3_ (50)	0.60 ± 0.09 c	0.58 ± 0.14 c
	1-MCP + GA_3_ (50)	0.77 ± 0.01 b	0.56 ± 0.00 c
	GA_3_ (100)	0.41 ± 0.02 d	0.37 ± 0.05 d
	1-MCP + GA_3_ (100)	0.76 ± 0.00 b	0.63 ± 0.00 b
Quercetin	Control	1.07 ± 0.05 d	0.76 ± 0.01 c
	1-MCP	1.41 ± 0.00 a	1.34 ± 0.00 a
	GA_3_ (50)	1.27 ± 0.05 bc	0.95 ± 0.01 b
	1-MCP + GA_3_ (50)	1.15 ± 0.04 c	0.97 ± 0.02 b
	GA_3_ (100)	0.94 ± 0.03 de	0.89 ± 0.03 b
	1-MCP + GA_3_ (100)	0.96 ± 0.00 de	0.92 ± 0.00 b

*: Values are mean value±standard deviation. *n* = 3 for the phenolic compounds. The difference among the means indicated with the same lowercase letter in columns was not significant (*p* ≤ 0.05).

## Data Availability

Data is contained within the article.
